# The fission yeast SUMO-targeted ubiquitin ligase Slx8 functionally associates with clustered centromeres and the silent mating-type region at the nuclear periphery

**DOI:** 10.1242/bio.061746

**Published:** 2024-12-30

**Authors:** Shrena Chakraborty, Joanna Strachan, Kamila Schirmeisen, Laetitia Besse, Eve Mercier, Karine Fréon, Haidao Zhang, Ning Zhao, Elizabeth H. Bayne, Sarah A. E. Lambert

**Affiliations:** ^1^ Institut Curie, Université PSL, CNRS UMR3348, 91400 Orsay, France; ^2^ Université Paris-Saclay, CNRS UMR3348, 91400 Orsay, France; ^3^ Institute of Cell Biology, School of Biological Sciences, University of Edinburgh, Edinburgh EH9 3FF, UK; ^4^ Institut Curie, Université PSL, CNRS UAR2016, Inserm US43, Université Paris-Saclay, Multimodal Imaging Center, 91400 Orsay, France; ^5^ Equipe Labélisée Ligue Nationale Contre le Cancer, 91400 Orsay, France

**Keywords:** SUMO-targeted ubiquitin ligase, Slx8, SUMO, Centromere, Heterochromatin, Gene silencing

## Abstract

The SUMO-targeted ubiquitin ligase (STUbL) family is involved in multiple cellular processes via a wide range of mechanisms to maintain genome stability. One of the evolutionarily conserved functions of STUbL is to promote changes in the nuclear positioning of DNA lesions, targeting them to the nuclear periphery. In *Schizossacharomyces pombe*, the STUbL Slx8 is a regulator of SUMOylated proteins and promotes replication stress tolerance by counteracting the toxicity of SUMO conjugates. In order to study the dynamic dialectic between ubiquitinylation and SUMOylation in the nuclear space of the *S. pombe* genome, we analyzed Slx8 localization. Unexpectedly, we did not detect replication stress-induced Slx8 foci. However, we discovered that Slx8 forms a single nuclear focus, enriched at the nuclear periphery, which marks both clustered centromeres at the spindle pole body and the silent mating-type region. The formation of this single Slx8 focus requires the E3 SUMO ligase Pli1, poly-SUMOylation and the histone methyl transferase Clr4 that is responsible for the heterochromatin histone mark H3-K9 methylation. Finally, we established that Slx8 promotes centromere clustering and gene silencing at heterochromatin domains. Altogether, our data highlight evolutionarily conserved and functional relationships between STUbL and heterochromatin domains to promote gene silencing and nuclear organization.

## Teaser

**Summary:** The SUMO-targeted ubiquitin ligase Slx8 associates with heterochromatin loci enriched at the nuclear periphery and promotes gene silencing and nuclear organization in the yeast *Schizossacharomyces pombe*.

## INTRODUCTION

The nuclear architecture and the 3D genome organization have emerged as important regulation layers of genome maintenance, contributing to numerous DNA-associated transactions such as chromosome segregation, transcription and DNA repair (Misteli and Soutoglou, 2009). Chromatin displays functional compartmentalization: while gene-rich, transcriptionally active chromatin tends to localize to the interior of the nucleus, gene-poor, transcriptionally repressed heterochromatin is typically enriched at the nuclear periphery (NP), which is believed to provide a microenvironment favoring association of factors required for silencing (reviewed in Towbin et al., 2009). In many organisms, centromeres also cluster together at the NP, and this spatial organization has been shown to be important for promoting loading of centromeric proteins (Wu et al., 2022), silencing of repetitive elements (Padeken et al., 2013), and the prevention of micronuclei formation (Jagannathan et al., 2018). The stability of the genome is particularly vulnerable during the process of DNA replication since a broad spectrum of obstacles can jeopardize the progression of the replication machinery, resulting in fork stalling, collapse or breakage (Zeman and Cimprich, 2014). In several organisms, from yeast to flies and mammalian cells, DNA lesions, including double-strand break (DSB) and replication stress site, shift away from their initial nuclear compartment to associate with the NP. Such mobility of DNA lesions allows a spatial regulation of DNA repair processes to ensure optimal error-free repair outcome (reviewed in Lamm et al., 2021; Whalen and Freudenreich, 2020).

The NP is composed of a double-membrane nuclear envelope (NE) and multiple nuclear pore complexes (NPCs) embedded in the NE. In yeast, the spindle pole body (SPB), the functional macromolecular structure equivalent to the centrosome, is also embedded in the NE. Components of both the NE and the NPC have been reported as factors allowing anchorage of DNA lesions to the NP (reviewed in Whalen and Freudenreich, 2020). Although the mechanisms of relocation and anchorage differ depending on the type of DNA lesion and the cell cycle stage, an emerging common feature is the requirement for SUMOylation, for which homeostasis is critical to maintain genome integrity (Schirmeisen et al., 2021). SUMO (small ubiquitin-like modifier) is a post-translational modification present in all eukaryotic systems. SUMO is covalently attached to a target thanks to the coordinated activity of E2 and E3 SUMO ligases (reviewed in Chang et al., 2021). Target proteins can either be mono-SUMOylated on a single lysine residue or harbor multiple single SUMO modifications on several lysine residues, a type of poly-SUMOylation. Moreover, additional SUMO molecules can be covalently attached to the internal lysine of SUMO to form SUMO chains, another type of poly-SUMOylation. SUMOylation affects the activity, the localization and stability of modified targets, with SUMO chains often favoring protein degradation.

A key determinant of the fate of SUMOylated proteins is the SUMO-targeted E3 ubiquitin ligase (STUbL) family that recognizes SUMOylated proteins and attaches ubiquitin to them. STUbLs are involved in diverse molecular processes, including DNA repair and replication, both during unchallenged conditions and in response to genotoxic stresses (reviewed in Chang et al., 2021). STUbLs are characterized by a RING-type E3 ubiquitin ligase domain and one or several SUMO-interacting motifs (SIMs) to recognize SUMOylated substrates. Modification by STUbLs can target substrates for proteosomal degradation or mediate non-proteolytic functions. STUbLs act in specific environments, such as the NE, centromere, kinetochore or PML nuclear bodies in human cells. STUbLs have also been implicated in localizing DSBs and replication stress sites to the NP to promote DNA repair and fork restart (reviewed in Lamm et al., 2021; Whalen and Freudenreich, 2020). A seminal study in *Saccharomyces cerevisiae* (Sc) first showed that difficult-to repair DSBs and collapsed forks anchor to the NPC in a process requiring the ScSlx5-Slx8 STUbL that physically associates with the Nup84 complex, a component of the NPC (Nagai et al., 2008). Further studies established that the SUMOylation status of proteins bound to DSBs influences the target destination. For example, mono-SUMOylation allows S-phase DSBs to relocate to Mps3, a NE component, whereas poly-SUMOylation allows DSBs in G1 to associate with the NPC in STUbL-dependent manner, suggesting a specificity of STUbL for poly-SUMO chains (Horigome et al., 2014, 2016).

The target destination of replication stress sites described so far is the NPC. This includes forks stalled within telomeres sequences, at tri-nucleotides repeats, at a replication fork barrier (RFB) mediated by DNA-bound protein and forks stalled by global replication stress in human cells (Aguilera et al., 2020; Kramarz et al., 2020; Nagai et al., 2008; Pinzaru et al., 2020; Rivard et al., 2024; Su et al., 2015). In *S. cerevisiae*, forks stalled at expanded CAG repeats, anchor to the NPC in a process that requires the SIMs of Slx5 and mono-SUMOylation, since preventing poly-SUMOylation does not affect relocation to the NP (Su et al., 2015). The SUMOylation of the repair factors RPA, Rad52 and Rad59 is sufficient to trigger Slx5-dependent relocation to the NP, suggesting that Slx5 may recognize several SUMO particles covalently attached to distinct targets (Whalen et al., 2020). Targeting forks stalled at CAG repeats to the NPC allows the loading of the recombinase Rad51 and prevents the chromosomal fragility of CAG repeats.

In *Schizosaccharomyces pombe* (Sp), we have revealed a SUMO-based mechanism that allows the spatial regulation of the recombination-dependent replication (RDR) process, a mechanism that ensures the restart of arrested forks by homologous recombination (Kramarz et al., 2020). Forks arrested by the *RTS1*-RFB relocate to the NP to associate with the NPC in a process requiring SUMO chain formation and the SpSTUbL. In *S. pombe*, Rfp1 and Rfp2 are functional homologs of ScSlx5 but lack E3 activity. They recruit Slx8 through a RING-RING domain interaction to form a functional E3 ubiquitin ligase (Prudden et al., 2007, 2011). The absence of a functional SpSlx8 STUbL results in the accumulation of high-molecular weight (HMW) SUMO conjugates and sensitivity to genotoxic drugs that can be alleviated by the inactivation of the E3 SUMO ligase Pli1 and by preventing SUMO chain formation, suggesting that SpSTUbL has specificity in targeting poly-SUMOylated substrates (Kosoy et al., 2007; Nie et al., 2017; Prudden et al., 2007; Steinacher et al., 2013). We further established that the relocation of the RFB to the NP promotes RDR via two activities that are enriched in the NPC environment, namely the SUMO protease Ulp1 and the proteasome (Schirmeisen et al., 2024).

One of the unresolved questions in the field is to understand the dynamic crosstalk between SUMOylation and ubiquitination during the process of relocation of stressed forks and how such crosstalk is spatially segregated in the nuclear space. For example, both SUMOylation and STUbL activity are expected to occur at the site of replication stress before relocation to the NP. Indeed, the *Drosophila* STUbL Dgrn (for degringolade) is recruited at heterochromatic DSBs prior to relocation and after the action of E3 SUMO ligases (Ryu et al., 2015, 2016). To investigate the temporal and spatial dynamics of SpSlx8 by live-cell imaging in response to global replication stress, we generated a functional fusion protein Slx8-GFP, in a similar approach to the one employed to characterize damage-induced ScSlx5 foci (Cook et al., 2009) and SpUfd1 (for ubiquitin-fusion degradation protein) that physically interacts with STUbL (Køhler et al., 2013). We observed that Slx8-GFP did not form replication stress-induced foci, but a single discrete focus enriched at the NP in unstressed condition. Both SUMO chains and the E3 SUMO ligase Pli1 are necessary to sustain Slx8-GFP focus formation. Further cellular analysis established that Slx8-GFP focus marks heterochromatin domains positioned at the NP and in the SPB environment, including centromeres and the mating-type (*mat*) region. Both heterochromatin and anchoring of centromeres to SPB promotes Slx8-GFP focus. Finally, we provide functional evidence that Slx8 is actively involved in gene silencing and in the clustering of centromeres. Our results highlight functional and physical crosstalk between STUbL and heterochromatin to orchestrate the nuclear organization of specific domains.

## RESULTS

### Slx8-GFP forms a single nuclear focus in a SUMO chain-dependent manner

**Fig. 1.**
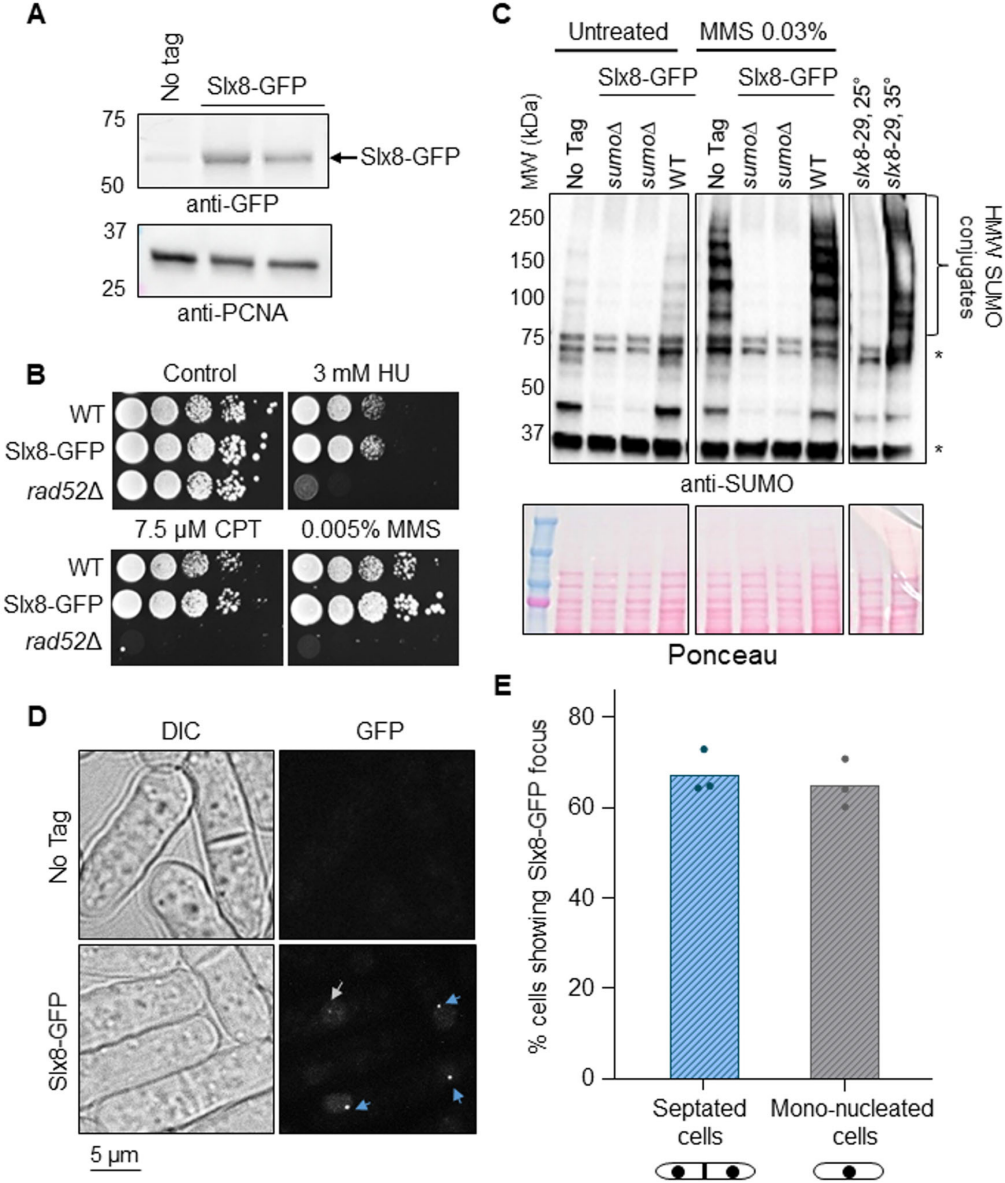
**Slx8-GFP forms a single focus in unstressed conditions.** (A) Expression of the endogenously GFP-tagged Slx8 fusion protein. An untagged WT strain (‘No tag’) was included as control for antibody specificity. PCNA was used as a loading control. Slx8-GFP has a molecular weight (MW) of 58 kDa. (B) Sensitivity of the indicated strains to indicated genotoxic drugs. Tenfold serial dilutions of exponential cultures were dropped on appropriate plates. HU, hydroxyurea; CPT, camptothecin; MMS, methyl methane sulfonate. (C) Expression of SUMO conjugates in the indicated strains and conditions. A strain deleted for *pmt3* gene that encodes the SUMO particle (*sumo*Δ) was added as control for antibody specificity. * indicates unspecific signal. A strain bearing the temperature-sensitive allele *slx8-29* was grown at permissive (25°C) and restrictive (32°C) temperature. HMW, high molecular weight. (D) Example of bright-field (left column, DIC) and GFP fluorescence (right column) images of cells expressing the endogenous Slx8-GFP fusion protein in indicated strains. Blue and gray arrows indicate Slx8-GFP foci in septated and mono-nucleated cells, respectively. Scale bar: 5 µm. (E) Histogram plots showing the percentage of septated and mono-nucleated cells with nuclear Slx8-GFP foci. *P*-value was calculated by two-tailed *t*-test (ns, non-significant). Dots represent values obtained from three independent biological experiments. At least 200 nuclei were analyzed for each strain and cell type.

**Fig. 2.**
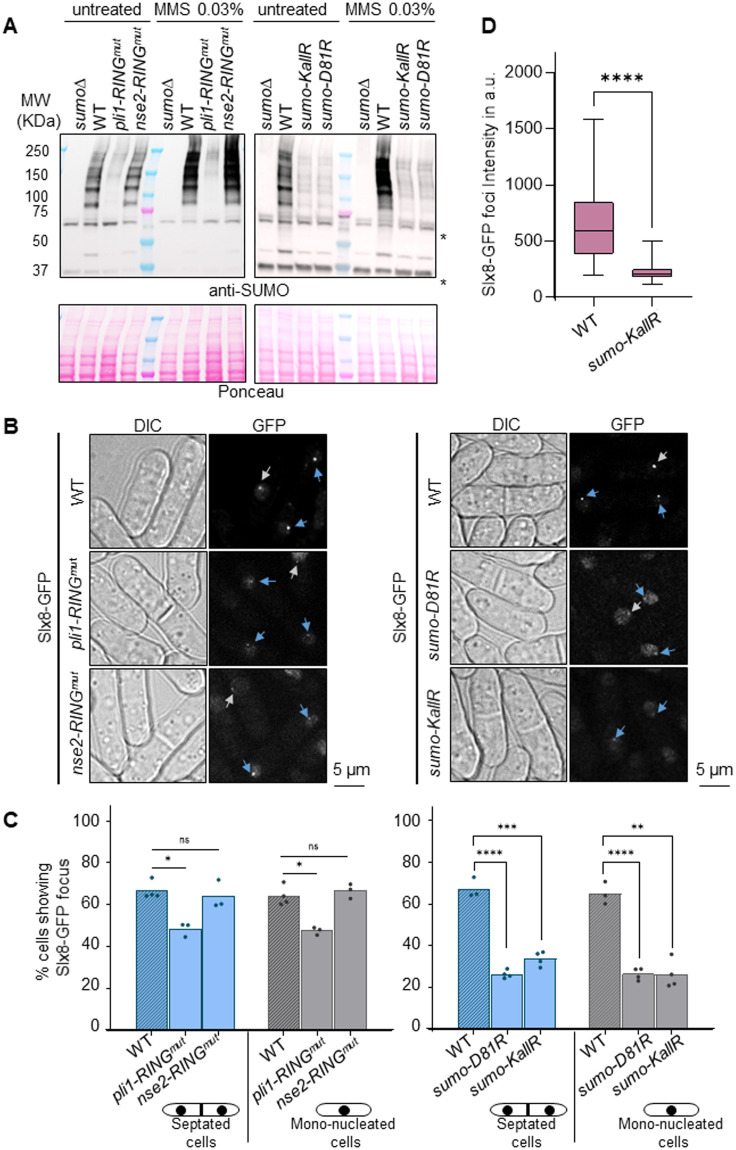
**SUMOylation promotes the formation of Slx8-GFP foci.** (A) Expression of SUMO conjugates in the indicated strains (expressing Slx8-GFP) and conditions. A strain deleted for *pmt3* gene that encodes the SUMO particle (*sumo*Δ) was added as control for antibody specificity. * indicates unspecific signal. (B) Example of bright-field (DIC) and GFP fluorescence images in indicated strains expressing Slx8-GFP. Blue and gray arrows indicate Slx8-GFP foci in septated and mono-nucleated cells, respectively. Scale bars: 5 µm. (C) Histogram plots showing the percentage of septated and mono-nucleated cells with nuclear Slx8-GFP foci in the indicated strains. *P*-value was calculated by two-tailed *t*-test (****P≤0.0001; ***P≤0.001; **P≤0.01; *P≤0.05; ns, non-significant). Dots represent values obtained from independent biological experiments. At least 200 nuclei were analyzed for each strain and cell type. (D) Box-and-whisker plots of Slx8-GFP intensity (mean fluorescence intensity) in the indicated strains. Boxes represent the 25/75 percentile, black lines indicate the median, the whiskers indicate the 5/95 percentile. *P*-value was calculated by Mann–Whitney *U*-test (****P≤0.0001). Values were obtained from at least two independent biological experiments. At least 60 nuclei were analyzed for each strain.

To investigate the spatial dynamics of SUMO conjugates prone to STUbL-dependent processing, Slx8 was C-terminally tagged with GFP, and Slx8-GFP functionality was established based on resistance to genotoxic stress (Fig. 1A,B). To further confirm that the GFP tag did not interfere with Slx8 function, we analyzed global SUMO conjugates by immuno-blotting. We observed an accumulation of HMW SUMO conjugates in the strain bearing the temperature-sensitive *slx8-29* allele when grown at the restrictive temperature (35°C), but not at the permissive temperature (25°C), indicating defective processing of SUMO conjugates in the absence of a functional Slx8 pathway, as expected (Fig. 1C) (Nie et al., 2017). None of these HMW SUMO conjugates were detected in wild-type (WT) or Slx8-GFP-expressing strains in untreated conditions, whereas they accumulated similarly in both strains upon cell exposure to methyl methane sulfonate (MMS), an alkylating agent known to induce global SUMOylation (Fig. 1C) (Nie et al., 2017). These results confirm that the Slx8-GFP fusion protein is functional. Then, we performed live-cell imaging and observed that Slx8-GFP formed a single bright focus in most septated cells, which correspond to the bulk of S-phase, and mono-nucleated cells, which mainly correspond to G2 cells (Fig. 1D,E). To address the link between this single Slx8-GFP focus and SUMO metabolism, we investigated the role of the two E3 SUMO ligases known in *S. pombe*: the SUMO chain-modified Pli1 and Nse2 that is dedicated to DNA damage response (Andrews et al., 2005; Prudden et al., 2011; Steinacher et al., 2013). We made use of point mutations in the RING domain of each protein to abolish the E3 SUMO ligase activity. Global SUMOylation was considerably reduced in cells expressing the mutated form Pli1-RING^mut^ (Pli1-C321S-H323A-C326S), compared to WT, and no MMS-induced SUMO conjugates were detected (Fig. 2A), consistent with Pli1 being responsible for most of global SUMOylation. In contrast, the global level of SUMO-conjugates was unaffected in cells expressing the mutated form Nse2-RING^mut^ (Nse2-C195S-H197A), despite this mutation rendering cells sensitive to genotoxic agents (Fig. 2A; Fig. S1A), as previously reported (Andrews et al., 2005; Prudden et al., 2011). Of note, the combination of Slx8-GFP with either Pli1-RING^mut^ or Nse2-RING^mut^ did not aggravate the cell sensitivity to genotoxic agents, further confirming the functionality of Slx8-GFP (Fig. S1A). Interestingly, the Slx8-GFP focus was less frequently observed in S- and G2-phase of *pli1-RING^mut^* cells, whereas no differences were detected in *nse2-RING^mut^* cells, compared to WT (Fig. 2B,C). Of note, the expression level of Slx8-GFP in *pli1-RING^mut^* and *nse2-RING^mut^* was similar to WT, excluding that the lack of Slx8-GFP focus resulted from an expression defect (Fig. S1B,C). We were unable to address the potential overlapping role of Nse2 and Pli1 in promoting Slx8-GFP focus formation, since spores harboring both *pli1-RING^mut^* and *nse2-RING^mut^* alleles were unviable. We concluded that the SUMO E3 ligase Pli1, which is responsible for global SUMOylation, sustains the formation of the single nuclear Slx8-GFP focus.

Next, we asked which type of SUMOylation contributes to the formation of the Slx8-GFP focus. We could not employ the strain harboring the deletion of the SUMO particle (*pmt3Δ*, hereafter SUMOΔ), since this strain shows frequent nuclear deformation. Instead, we employed a strain expressing SUMO-KallR, in which all internal lysine is mutated to arginine to prevent SUMO chain formation (Kramarz et al., 2020) and a strain expressing SUMO-D81R that allows mono- and di-SUMOylation to occur but impairs the chain-propagating role of Pli1 (Prudden et al., 2011). As expected, global SUMOylation was massively reduced in strains expressing SUMO-KallR and SUMO-D81R, even upon MMS treatment, compared to WT (Fig. 2A). Consistently, the frequency of cells showing a single nuclear Slx8-GFP focus was reduced by almost two-thirds in SUMO-KallR and SUMO-D81R cells, compared to WT (Fig. 2B,C), indicating that SUMO-chains are critical determinants of Slx8-GFP focus formation. Of note, Slx8-GFP expression level was only slightly reduced (by ∼20%) in SUMO-D81R, an insufficient reduction to explain the lack of two-thirds of the foci (Fig. S2). In addition to being less frequently formed, Slx8-GFP foci were three to four times less intense in SUMO-KallR cells, compared to WT (Fig. 2D). We concluded that the formation of the single nuclear Slx8-GFP focus requires SUMO chain formation and the SUMO-chain modified E3 ligase Pli1, suggesting that it marks SUMO conjugates at specific nuclear regions.

### Slx8-GFP does not form supernumerary foci in response to replication stress

**Fig. 3.**
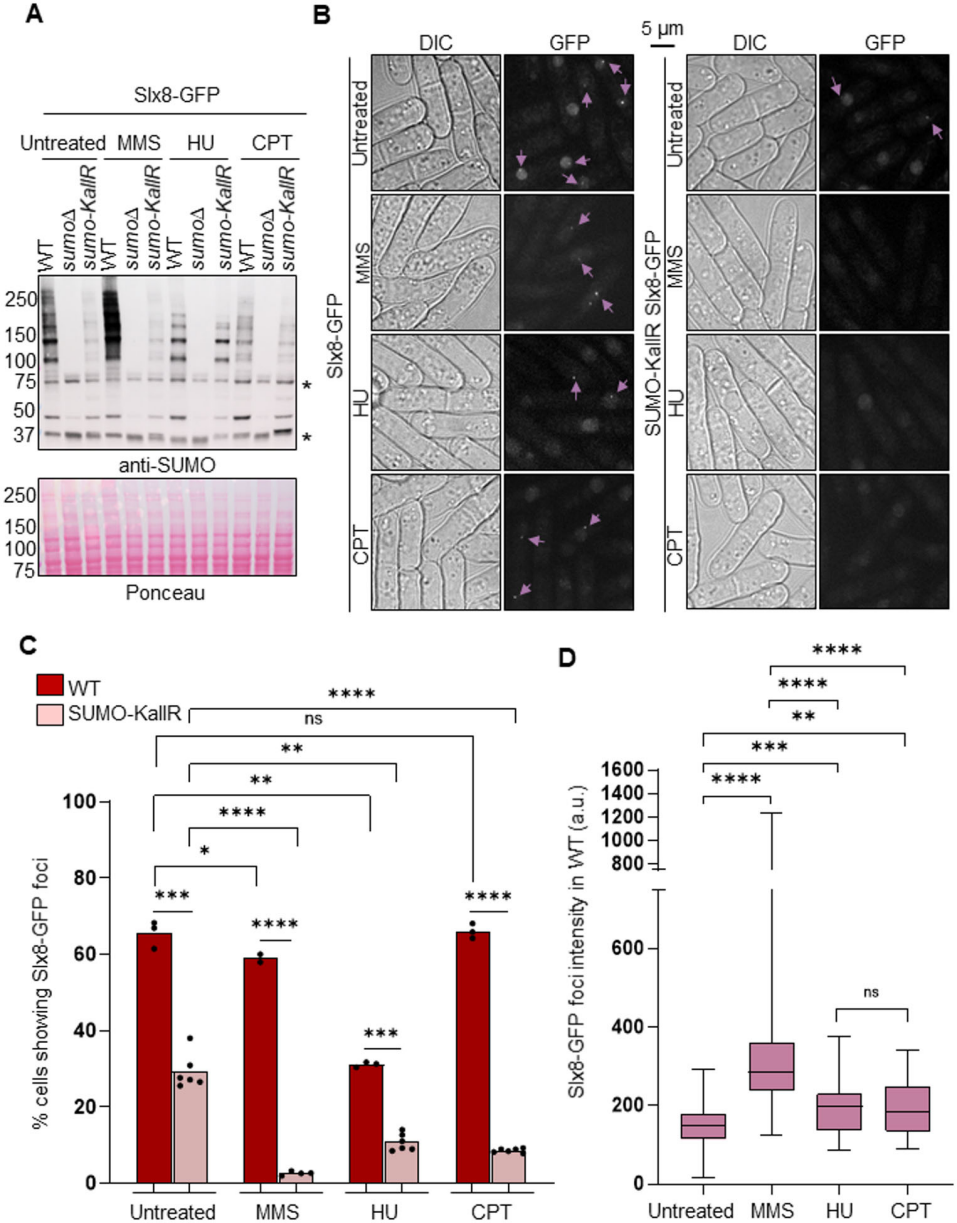
**Genotoxic stress does not lead to supernumerary Slx8-GFP foci.** (A) Expression of SUMO conjugates in the indicated strains (expressing Slx8-GFP) and conditions. A strain deleted for *pmt3* gene that encodes the SUMO particle (*sumo*Δ) was added as control for antibody specificity. * indicates unspecific signal. Strains were treated with genotoxic drugs before the extraction of proteins. HU, hydroxyurea (20 mM, 4 h); CPT, camptothecin (40 µM, 4 h); MMS, methyl methane sulfonate (0.03%, 3 h). (B) Example of bright-field (DIC) and GFP fluorescence images in the indicated strains and conditions. Genotoxic stresses were generated as in A. Pink arrows indicate cells harboring nuclear Slx8-GFP foci. Scale bar: 5 µm. (C) Histogram plots showing the percentage of cells with nuclear Slx8-GFP foci in the indicated strains and conditions. *P*-value was calculated by two-tailed *t*-test (****P≤0.0001; ***P≤0.001; **P≤0.01; *P≤0.05; ns, non-significant). Dots represent values obtained from two independent biological experiments. At least 200 nuclei were analyzed for each strain and treatment condition. (D) Box-and-whisker plots of Slx8-GFP intensity (mean fluorescence intensity) in the indicated strains and conditions. Boxes represent the 25/75 percentile, black lines indicate the median, the whiskers indicate the 5/95 percentile. *P*-value was calculated by Mann–Whitney *U*-test (****P≤0.0001; ***P≤0.001; **P≤0.01; ns, non-significant). Values were obtained from two independent biological experiments. At least 60 nuclei were analyzed for each strain and treatment condition.

Having establish that Slx8-GFP marks specific nuclear regions in a SUMO-dependent manner, we investigated if Slx8-GFP forms DNA damage-induced foci, as reported for ScSlx5 (Cook et al., 2009). Treatment with MMS, but not with hydroxyurea (HU; an inhibitor of the ribonucleotide reductase leading to a depletion of the dNTP pool and stalled replication fork) or camptothecin (CPT; an inhibitor of the topoisomerase I leading to collapsed replication fork), resulted in a marked accumulation of SUMO conjugates (Fig. 3A). Whatever the replication-blocking agent used, no additional DNA damage-induced Slx8 foci could be detected in our microscopy setup on living cells, even in condition of MMS-induced accumulation of SUMO conjugates (Fig. 3A-C). Surprisingly, HU treatment resulted in a 50% reduction in cells showing a single Slx8 focus in WT cells. It is worth noting that, despite the absence of supernumerary Slx8-GFP foci, the intensity of the single Slx8-GFP focus increased significantly upon exposure to genotoxic stresses, particularly after MMS treatment, compared with the untreated condition (Fig. 3D). Furthermore, the frequency of cells showing a single Slx8-GFP focus was severely reduced in SUMO-KallR cells after treatment with replication blocking agents (Fig. 3B,C), suggesting that SUMO chains become more critical for maintaining the Slx8 GFP focus under replication stress conditions. Although we observed a slight decrease in Slx8-GFP expression in WT and SUMO-KallR cells in response to treatments (Fig. S3), the extent of variation seems insufficient to explain the disappearance of Slx8-GFP foci. We concluded that Slx8-GFP cannot serve as a readout of damage-induced SUMO chain formation but that the behavior of the single Slx8-GFP focus is modulated by replication stress in a SUMO chain-dependent manner.

### The single nuclear Slx8-GFP focus marks centromere and the *mat* region at the nuclear periphery

**Fig. 4.**
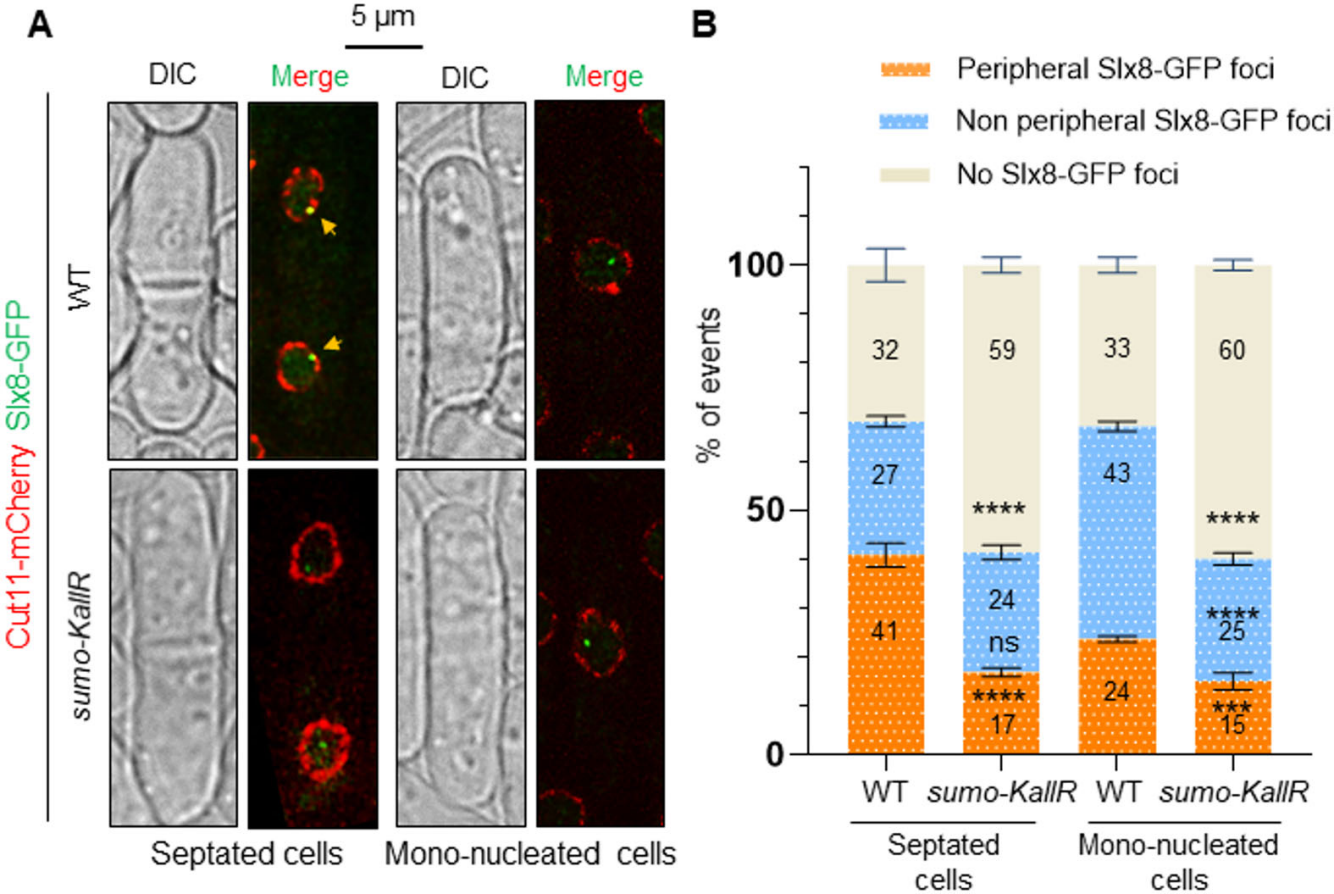
**Slx8-GFP focus is enriched at the nuclear periphery.** (A) Representative cell images of cells expressing Cut11-mCherry (red) and Slx8-GFP (green) in septated and mono-nucleated cells of indicated strains. The nuclear periphery is visualized via Cut11-mCherry. Yellow arrows indicate colocalization events. Scale bar: 5 µm. (B) Stacked bar charts showing the frequency of colocalization between Slx8-GFP and Cut11-mCherry in septated and mono-nucleated cells of indicated strains. Individual bars represent 100% of events; numbers indicate the % of each category (peripheral Slx8-GFP foci colocalizing with Cut11-mCherry in orange, non-peripheral Slx8-GFP foci in blue, absence of Slx8-GFP foci in cream-white). *P*-value was calculated by two-tailed *t*-test (****P≤0.0001; ***P≤0.001; ns, non-significant). Bars indicate mean±s.d. Values were obtained from two independent biological experiments. At least 200 nuclei were analyzed for each strain and cell type.

The analysis of cell images revealed that the single Slx8-GFP focus in untreated condition was often positioned at the periphery of the nucleus. To confirm this, we asked how frequently Slx8-GFP foci colocalize with Cut11-mCherry, a component of the NPC that marks the NP. We found that the nuclear Slx8-GFP focus, where visible, was positioned at the NP in ∼65% of WT S-phase cells (septated cells) and this frequency dropped to ∼35% in WT G2 cells (mono-nucleated cells) (Fig. 4A,B). Interestingly, this peripheral nuclear positioning in S-phase dropped to ∼35% in cells expressing SUMO-KallR. We concluded that most Slx8 foci are enriched at the NP and that SUMO chains contribute to Slx8-GFP focus formation and positioning at the NP during S-phase.

**Fig. 5.**
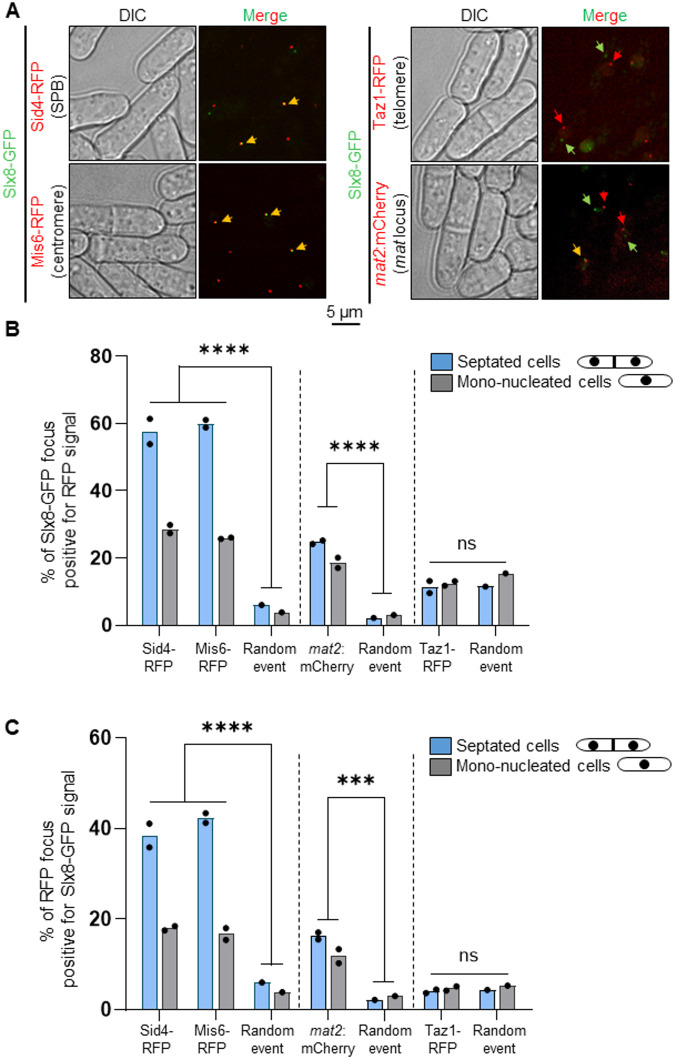
**Slx8-GFP focus marks the SPB, centromere and the mating-type locus.** (A) Representative cell images of strains expressing Slx8-GFP and either Sid4-RFP (a SPB marker) or Mis6-RFP (a kinetochore marker) or Taz1-RFP (a telomere marker), or harboring the endogenous *mat2* locus tagged with a LacO array bound by LacI-Mcherry (*mat2:mCherry*). Red, green and yellow arrows indicate RFP, GFP and colocalization events, respectively. Scale bar: 5 µm. (B,C) Histogram plots showing the percentage of colocalization events between Slx8-GFP and the above described markers. *P*-value was calculated by Brown-Forsythe and Welch ANOVA test (****P≤0.0001; ***P≤0.001; ns, non-significant). Dots represent values obtained from two independent biological experiments. At least 200 nuclei were analyzed for each strain and cell type.

The peripheral nuclear location of the single Slx8-GFP focus suggests that Slx8 associates with specific components and/or chromosomal regions known to be at the NP. During interphase, the *S. pombe* chromosomes are arranged in a Rabl-like configuration in which the three centromeres are clustered adjacent to the SPB embedded in the NE, while telomeres form discrete foci clustered at the NP at the opposing hemisphere of the nucleus (Mizuguchi et al., 2015). In addition, the heterochromatin domain of the sexual mating locus (hereafter *mat* region), that contains the silent *mat2* and *mat3* loci, is also positioned at the NP nearby the SPB. We thus addressed if Slx8-GFP localizes with markers of centromere (Mis6-RFP, a kinetochore component), SPB (Sid4-RFP) and telomere (Taz1-RFP) and the *mat* region (using a strain harboring a *LacO* array integrated nearby the *mat* locus, bound by the fluorescent repressor LacI-mCherry) (Fig. 5A). During S-phase (in septated cells), the nuclear Slx8-GFP focus colocalized with Sid4-RFP and Mis6-RFP in ∼60% of cells showing a Slx8-GFP focus, whereas a colocalization event with the *mat* region was observed in ∼20% of the cells (Fig. 5B). Such nuclear positioning appeared highly significant compared to random colocalization events. Although less pronounced, the Slx8-GFP focus significantly overlapped with the centromere, SPB and *mat* region in G2 cells (mono-nucleated cells). In contrast to the Slx8-GFP focus, all cells exhibited a single Sid4-RFP and Mis6-RFP focus, or a single LacI-mCherry dot marking the *mat* region (Fig. 5A). We found that the centromere and SPB are positively associated with Slx8 in 40% of S-phase cells and in 20% of G2 cells, whereas the *mat* region associated with Slx8 in ∼15-18% of S- and G2-phase cells (Fig. 5C). In contrast, no colocalization above random events was detected between Slx8-GFP and Taz1-marked telomeres foci. We concluded that, for the most part, the Slx8-GFP focus positioned at the NP marks clustered centromeres, the SPB and the *mat* region.

### Heterochromatin and centromeres clustering at SPB sustain Slx8-GFP focus formation

**Fig. 6.**
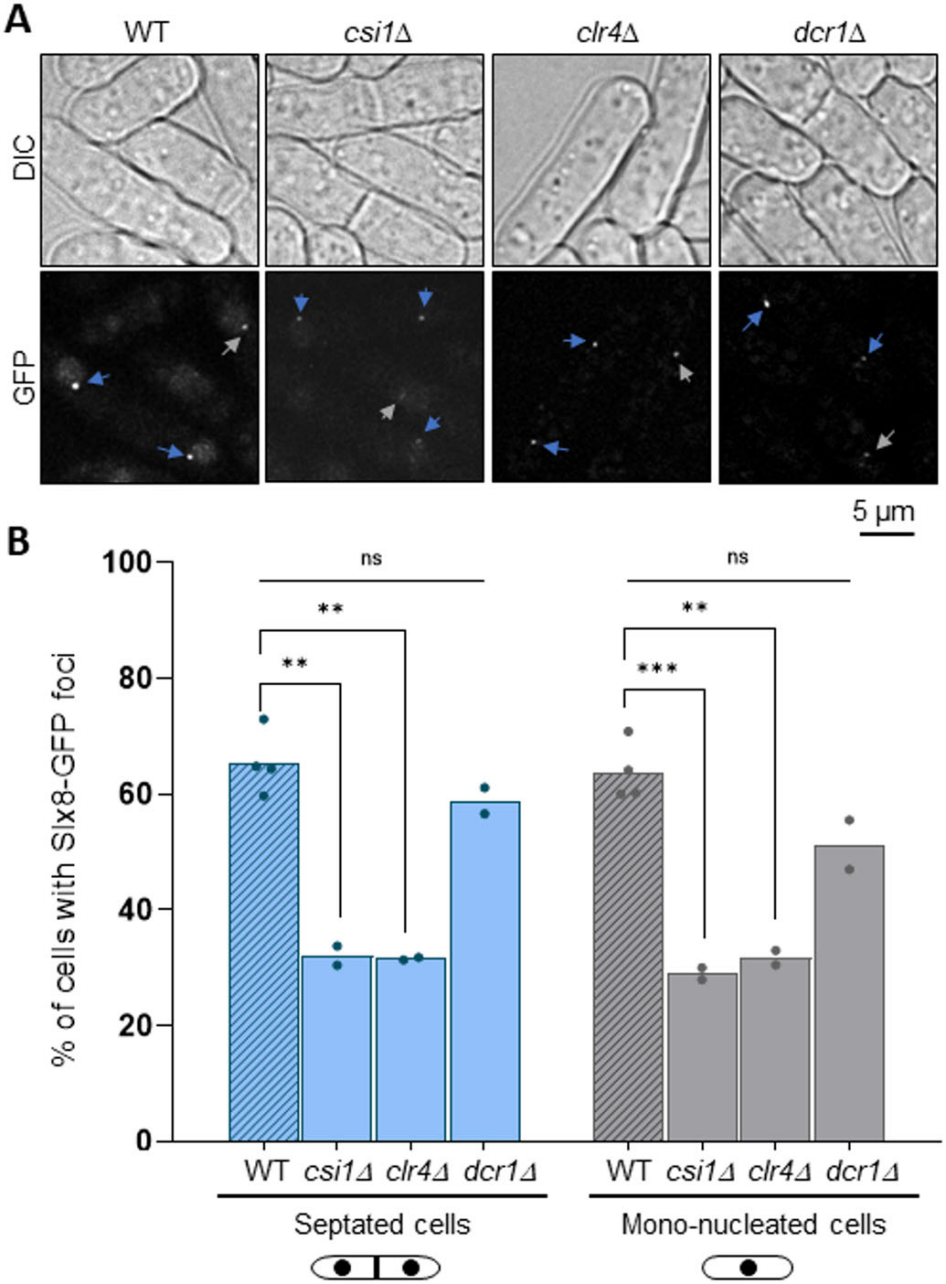
**Heterochromatin and centromere clustering promote Slx8-GFP focus formation.** (A) Example of bright-field (top row, DIC) and GFP fluorescence (bottom row) images of cells expressing Slx8-GFP in the indicated strains. Blue and gray arrows indicate Slx8-GFP foci in septated and mono-nucleated cells, respectively. Scale bar: 5 µm. (B) Histogram plots showing the percentage of septated and mono-nucleated cells with nuclear Slx8-GFP foci. *P*-value was calculated by two-tailed *t*-test (***P≤0.001; **P≤0.01; ns, non-significant). Dots represent values obtained from two independent biological experiments. At least 200 nuclei were analyzed for each strain and cell type.

Slx8-GFP marks the SPB environment and associated chromosomal regions such as centromeres and *mat* region, both being enriched for heterochromatin that ensures gene silencing. Therefore, we asked if heterochromatin formation and centromere clustering are required to ensure the formation of a single Slx8-GFP focus. We observed that, in the absence of Clr4, the histone methyl-transferase that promotes H3-K9 methylation, a hallmark of heterochromatin and gene silencing (Nakayama et al., 2001; Rea et al., 2000), the frequency of Slx8-GFP focus formation was reduced by twofold (Fig. 6A,B). In contrast, no effect was observed in the absence of Dicer (Dcr1), a component of the RNA interference (RNAi) machinery promoting the establishment of heterochromatin, but with only a partial role in maintenance. These results indicate that H3K9 methylation, but not RNAi, is required to promote the formation of the nuclear Slx8-GFP focus. We also investigated the role of centromere clustering. Csi1 is a key factor that provides a physical link between kinetochores and SPB associated proteins. The lack of Csi1 leads to a severe defect in centromere clustering (Hou et al., 2012) and resulted in a twofold reduction in the frequency of the Slx8-GFP focus (Fig. 6A,B). Of note, the expression of Slx8-GFP was not affected in the absence of Csi1, Clr4 or Dcr1, indicating that the decreased in the frequency of Slx8-GFP foci is not caused by variation in expression level (Fig. S4). Thus, both heterochromatin formation and centromere clustering contribute to Slx8-GFP focus formation.

### Slx8 promotes centromere clustering and gene silencing

**Fig. 7.**
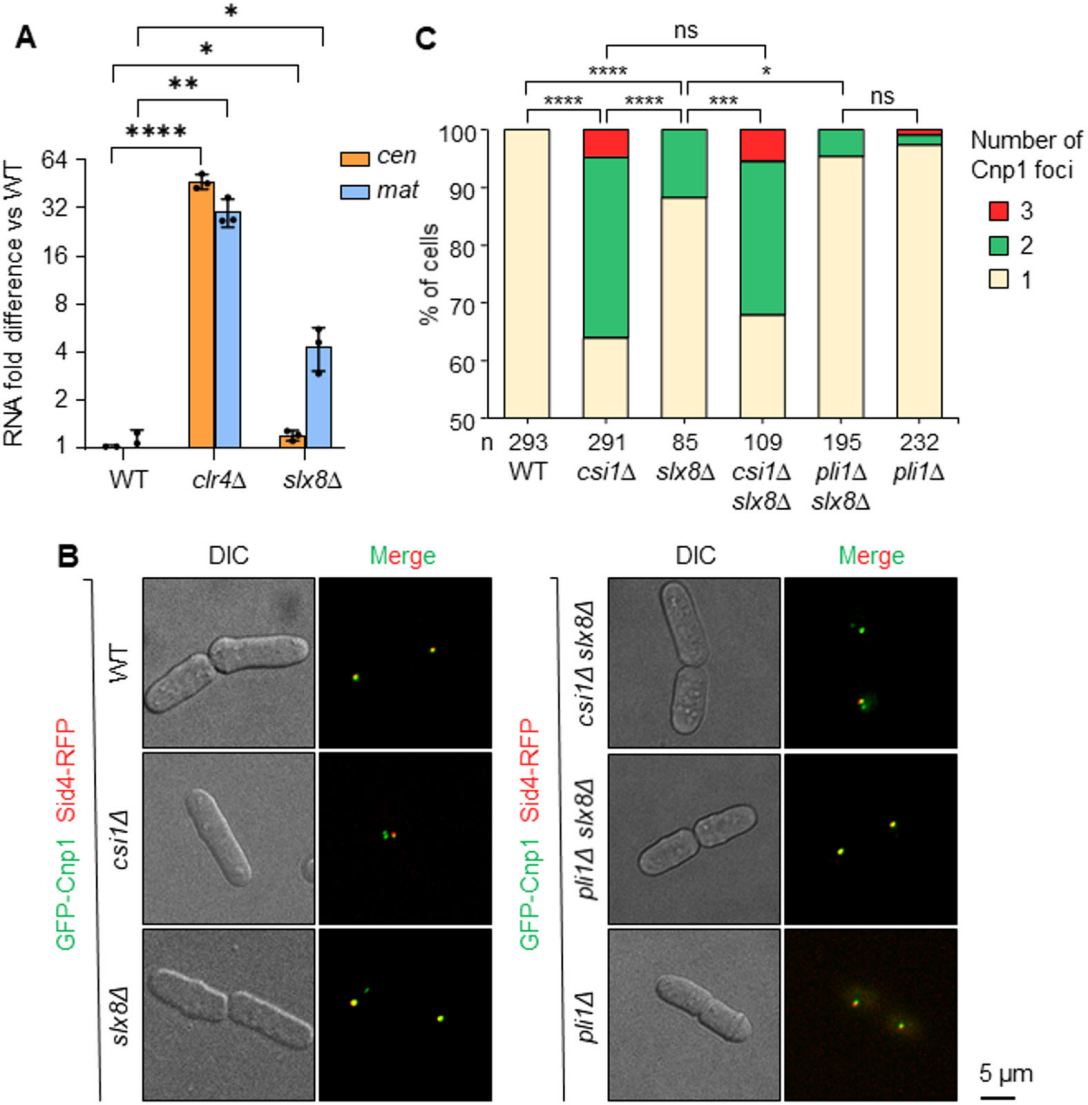
**Slx8 promotes heterochromatic silencing and centromere clustering.** (A) RT-qPCR analysis of pericentromere (*cen[dg]*) and mating-type locus (*mat*) transcript levels, normalized to control transcript *act1^+^*. Data are plotted as fold difference relative to wild-type, on log_2_ scale. *P*-value was calculated by two-tailed *t*-test (****P≤0.0001; **P≤0.01; *P≤0.05). Values are mean±s.d. from three independent experiments. (B) Representative images of cells expressing GFP–Cnp1 (centromere marker) and Sid4–RFP (SPB marker). Scale bar: 5 µm. (C) Stacked bar charts showing the percentage of cells displaying one, two or three Cnp1 foci, based on analysis of *n* nuclei. *P*-value was calculated by chi-squared test (****P≤0.0001; ***P≤0.001; *P≤0.05; ns, non-significant).

Finally, we tested whether Slx8 functions to promote heterochromatic silencing and centromere clustering. To assess silencing, we performed RT-qPCR analysis of transcripts from the heterochromatic pericentromere (*cen[dg]*) and silent mating-type (*mat*) regions. Such transcripts accumulate at very low levels in WT cells, but much higher levels in the absence of factors such as Clr4 required for heterochromatin assembly. Interestingly, we also observed a small, but significant, increase in the accumulation of transcripts from both the pericentromere and the *mat* locus in cells lacking Slx8, consistent with Slx8 functionally contributing to silencing in these regions (Fig. 7A). To assess centromere clustering, we performed live-cell imaging on cells expressing GFP–Cnp1 (*S. pombe* CENP-A, the centromere-specific histone variant) to visualize centromeres, together with Sid4–RFP as a marker of the SPB. Whereas WT cells consistently display a single GFP–Cnp1 focus, representing three clustered centromeres, adjacent to the SPB, absence of Csi1 results in ∼35% of cells showing more than one GFP–Cnp1 focus, indicative of defective clustering. Strikingly, the lack of Slx8 also resulted in a significant clustering defect, with ∼12% of cells displaying more than one GFP–Cnp1 focus (Fig. 7B,C). An epistatic phenotype was seen for *slx8*Δ *csi1*Δ double mutant cells, which displayed clustering defects comparable to those in the *csi1*Δ single mutant, suggesting that Slx8 may function in the same pathway as Csi1. Deletion of the SUMO ligase Pli1 largely suppressed the clustering defect associated with absence of Slx8, consistent with it arising as a result of excess SUMOylation. We conclude that localization of Slx8 in the vicinity of the SPB both depends on, and contributes to, heterochromatin integrity and centromere clustering.

## DISCUSSION

STUbL proteins play diverse roles throughout the cell cycle to protect against genome instability. Here, we revealed that the fission yeast STUbL Slx8 functions mainly in the SPB environment in a SUMO-dependent manner to help ensure centromere clustering and gene silencing at heterochromatic domains. These data are consistent with SUMOylation of centromeres being an important mediator of centromere identity, and indicate that Slx8 plays a critical role in regulating SUMO homeostasis in the nuclear space to safeguard centromere biology.

In several organisms, STUbL activities are linked to the maintenance of genome stability and resistance to DNA damage (reviewed in Chang et al., 2021). In *S. pombe*, Slx8 operates with Ufd1, a component of the Cdc48-Udf1-Npl4 that allows the extraction of ubiquitylated proteins from higher-order complexes (Køhler et al., 2013). Both physical and functional overlaps between Ufd1 and Slx8 have revealed that Slx8 helps in channeling SUMOylated proteins towards such extraction process. This mechanism is part of the DNA damage response as Ufd1 forms DNA damage-induced foci, colocalizing with SUMO at the nuclear periphery. Similarly, ScSlx5 forms damage-induced nuclear foci in a SUMO-dependent manner and colocalizes with DNA repair factors (Cook et al., 2009). On the other hand, discrepancies were reported regarding the nuclear distribution of ScSlx8, with either MMS-induced enrichment at the nuclear periphery, targeting to replication foci during unchallenged S-phase or targeting to intra-nuclear quality control (INQ) sites (Burgess et al., 2007; Gallina et al., 2015; Kumar et al., 2024; Nagai et al., 2008). Therefore, it was unanticipated that DNA damage does not lead to a redistribution of Slx8 to form specific DNA repair-associated foci. One possibility is that the amount of Slx8 recruited at site of DNA damage is below the level of detection offered in our cell microscopy condition.

Our observation of an Slx8 focus colocalizing with centromeres is consistent with several previous studies indicating that STUbLs reside and function at centromeres. In budding yeast, genome-wide binding analyses revealed centromeric enrichment of Slx5, but not of Slx8, and that cells lacking Slx5 or Slx8 display chromosome segregation defects (van de Pasch et al., 2013). Indeed, Slx5-Slx8 STUbL activity has been shown to be required for degradation of several centromere-associated substrates including cohesion subunit Mcd1 (D'Ambrosio and Lavoie, 2014), chromosome passenger complex (CPC) components Bir1 and Sli15 (Thu et al., 2016), and centromere-specific histone H3 variant Cse4^CENP-A^ (Cheng et al., 2017; Ohkuni et al., 2018), thereby promoting the proper specification and function of centromeres. Similarly, mammalian RNF4 has been implicated in regulating centromere and kinetochore assembly, functioning antagonistically with SUMO protease SENP6 to modulate levels of CENP-A assembly factor Mis18BP1 (Fu et al., 2019; Liebelt et al., 2019) and inner kinetochore protein CENP-I (Mukhopadhyay et al., 2010). In *S. pombe*, it has been reported previously that loss of Slx8 results in chromosome segregation defects, dependent on the SUMO ligase Pli1 (Steinacher et al., 2013); our findings that absence of Slx8 is associated with defects in both heterochromatic silencing and centromere clustering point to multifaceted roles of STUbL activity in supporting normal centromere function.

The formation of Slx8 focus is largely independent of the cell stage, as we observed similar frequency of cells exhibiting Slx8 focus in septated (bulk of S-phase) and mono-nucleated (G2-phase) cells (Fig. 1). Nonetheless, we noticed that the peripheral location of the Slx8 focus preferentially occurs during S-phase in a SUMO chain-dependent manner (Fig. 4B). Consistent with this, the maximum association of Slx8 with clustered centromeres and SPB was observed in septated cells, suggesting that DNA replication contributes to establish Slx8 focus formation. Of note, HU treatment, which led to synchronization of cells in G1/early S-phase, resulted in a severe decreased in the frequency of Slx8 foci (Fig. 3), suggesting that inhibition of DNA replication impairs Slx8 focus formation.

Heterochromatin is a key structural and regulatory component of centromeres in most eukaryotes, functioning to promote accurate chromosome segregation and silence repetitive DNA elements. Perturbation of SUMOylation has been linked to defects in heterochromatic silencing in several systems, including flies (Ninova et al., 2020), mammals (Marshall et al., 2010), and *S. pombe* (Shin et al., 2005). Moreover, large-scale studies in various organisms have identified heterochromatic regions including centromeres as SUMOylation hotspots (Cubeñas-Potts and Matunis, 2013; Ninova et al., 2023). Indeed, in *S. pombe*, proteomic analyses revealed that more than a third of SUMOylated proteins regulated by Slx8 and Ufd1 are proteins associated with centromeres or telomeres, including key heterochromatin regulators, the H3K9 methyltransferase Clr4 and anti-silencing factor Epe1 (Køhler et al., 2015). How SUMOylation impacts the function of these specific proteins has yet to be established. However, it has previously been shown that Epe1 is subject to ubiquitin-dependent cleavage and degradation that regulates its activity within heterochromatin domains (Braun et al., 2011) and in response to stress (Yaseen et al., 2022). It is thus tempting to speculate that Slx8 STUbL activity might contribute to Epe1 ubiquitination, and therefore that alleviation of heterochromatic silencing in *slx8*Δ cells could potentially be attributable, at least in part, to increased Epe1 activity. Since the localization of potential substrates such as Epe1 and Clr4 at centromeres and the *mat* locus is heterochromatin dependent (Isaac et al., 2007; Zofall and Grewal, 2006), this could help explain why Slx8 association with these regions is both dependent on, and required for, proper heterochromatin maintenance.

The phenomenon of centromere clustering has been observed in many eukaryotes and also appears to be important for normal centromere function, although the underlying mechanisms are not yet fully understood. In fission yeast, Csi1 plays an important role in tethering kinetochores to the SPB, and loss of this protein results in centromere declustering (Hou et al., 2012). We previously uncovered a role for SUMOylation in enhancing centromere clustering in conditions where Csi1 is absent, since removal of nucleoporin Nup132, which tethers SUMO protease Ulp1 to the NP, causes a SUMO-dependent rescue of clustering in *csi1*Δ cells (Strachan et al., 2023). This effect was found to be dependent on SUMOylation of the inner nuclear membrane protein Lem2, which acts in parallel with Csi1 to promote clustering, but independent of Slx8 activity. In contrast, here we show that Slx8 is required to maintain proper centromere clustering in otherwise WT cells. The relevant substrate(s) in this case have yet to be determined; however, our genetic data suggest that substrate(s) likely lie in the same pathway as Csi1, and therefore could potentially include, for example, Csi1 itself, or the interacting NE protein Sad1, both of which have been shown to be subject to SUMOylation (Køhler et al., 2015). In principle, Slx8 activity may be required either to temper the accumulation of SUMOylated proteins, or to actively promote protein extraction/turnover in a SUMO-dependent manner. However, we have shown previously that loss of the SUMO ligase Pli1 has only minimal effect on centromere clustering in this background (∼2.5% of *pli1*Δ cells displaying declustering, as compared to ∼12% of *slx8*Δ cells), whereas we confirm here that the clustering defects associated with absence of Slx8 are largely suppressed upon removal of Pli1. Thus, it is likely that Slx8 is primarily required to prevent the detrimental excess accumulation of SUMOylated substrates, and therefore to help maintain an optimal balance of SUMOylation needed to support normal centromere clustering.

SUMOylation has been found to influence the dynamics of telomere maintenance in *S. pombe* by controlling the activity of positive or negative regulators of telomerase (Xhemalce et al., 2004, 2007). In our microscopy analysis, we could not detect association between Slx8 and Taz1-marked telomeres. It is worth noting that, in budding yeast, telomeric factors are enriched for SUMO modifications upon telomere erosion (in the absence of telomerase), resulting in Slx5-Slx8-mediated relocation to the NPC to promote telomere length maintenance (Churikov et al., 2016). Therefore, it is possible that, without stress-inducing SUMOylation at telomeres, the association between Slx8 and telomere is below the limit of detection. Nonetheless, our results highlight that, in unchallenged conditions, Slx8 mainly acts at heterochromatic domains and centromeres to orchestrate the nuclear organization and functions of these specific domains.

## MATERIALS AND METHODS

### Standard yeast genetics and biological resources

Yeast strains used in this work are listed in Table S1. Gene deletion and tagging were performed by classical genetic techniques. To assess the sensitivity of chosen mutants to genotoxic agents, mid-log-phase cells were serially diluted and spotted onto yeast extract agar plates containing hydroxyurea (HU), methyl methanesulfonate (MMS) or campthotecin (CPT).

### Live-cell imaging

For snapshot microscopy, cells were grown in filtered Edinburgh's Minimal Medium containing glutamate (EMMg) to exponential phase, then centrifuged and resuspended in 500 µl of fresh EMMg. Then, 1 µl from the resulting solution was dropped onto a Thermo Fisher Scientific slide (ER-201B-CE24) covered with a thin layer of 1.4% agarose in filtered EMMg (Kramarz et al., 2020). Eleven *Z*-stack pictures (each *Z*-step of 200 nm) were captured using a Nipkow Spinning Disk confocal system (Yokogawa, CSU-X1-A1) mounted on a Nikon Eclipse Ti E inverted microscope, equipped with a 100× Apochromat TIRF oil-immersion objective (1.49 NA) and captured on sCMOS Prime 95B camera (Photometrics) operated through MetaMorph^®^ software (Molecular Devices). The GFP proteins were excited with a 488 nm (Stradus^®^, Vortran Laser Technology, 150 mW) laser, while RFP and mCherry proteins were excited with a 561 nm (Jive™, Cobolt, 100 mW) laser. A quad-band dichroic mirror (405/488/568/647 nm, Semrock) was used in combination with single band-pass filters of 525/50 or 630/75 for the detection of GFP and RFP/mCherry, respectively. Fluorescence and bright-field 3D images were taken at every 0.2 µm by acquiring one wavelength at a time. Exposure time for the GFP channel was 500 ms, for RFP was 300 ms and for mCherry was 600 ms. During the imaging, the microscope was set up at 25°C. For all the experiments the Gataca Live SR module (Gataca Systems), implemented on the Spinning Disk confocal system, was used to generate super-resolution images. All image acquisition was performed at the PICT-IBiSA Orsay Imaging facility of Institut Curie.

### Image analysis

Images were mounted and analyzed with Fiji software (Schindelin et al., 2012). First, the 3D z series were converted into 2D projections based on maximum-intensity values to produce the image with merged stacks. Since Slx8 is a low-abundant protein, with a high nuclear background, the quantification of Slx8 foci was performed using a noise tolerance threshold value of 50 (Maxima) from Fiji. This was decided after comparing different Maxima values in order to detect foci versus random background noise. Once the threshold was applied, the foci could be manually counted by selecting them as detected by the software. All experiments have been analyzed with the same Maxima value in this report. For quantification of the percentage of colocalization between Slx8 and other markers, the same as above was done onto the GFP channel to first annotate the Slx8 foci above the ‘set’ threshold. In a separate window, the GFP and RFP/mCherry channels with different stacks were merged together followed by manually analyzing the colocalization of the green and red foci signal at each stack. Maxima was not applied to the RFP/mCherry channel because the foci detection was clear and obvious with no nuclear background noise. The probability of a random event for the colocalization experiments were performed by using the 180° transform tool in Fiji for the RFP/mCherry marker, followed by merge with the normal Slx8 GFP stacks (without the 180° transform). Consequently, analysis of colocalization between the green and red foci signal at each stack in this setting provided the number of random colocalization events possible in each given field. This value is referred to as the ‘random event’ that provides a threshold to calculate the possibility of significant colocalization events as compared to random events.

### Centromere clustering analysis

For clustering analysis, cells expressing GFP–Cnp1 and Sid4–RFP were grown in YES to exponential phase, then centrifuged and resuspended in 30 µl YES. Then, 4 µl of the resulting cell suspension was mixed with 6 µl of 1% low-melting point agarose, and imaging was performed at 25°C using a Nikon Ti2 inverted microscope, equipped with a 100×1.49 NA Apo TIRF objective and a Teledyne Photometrics Prime 95B camera. Images were acquired with NIS-elements (version 5.1), with *Z*-stacks taken at 250 nm intervals. Maximum intensity *Z*-projections were made in ImageJ. Manual quantification of the number of GFP foci per cell was performed to determine the proportions of cells displaying centromeres ‘clustered’ (one GFP–Cnp1 focus) versus ‘unclustered’ (two or three GFP–Cnp1 foci).

### Whole-protein extract analysis

Aliquots of 1×10^8^ cells were collected and disrupted by bead beating in 1 ml of 20% trichloroacetic acid (TCA; Sigma-Aldrich, T9159). Pellets of denatured proteins were washed with 1 M Tris pH 8 and resuspended in 2× Laemmli buffer (62.5 mM Tris pH 6.8, 20% glycerol, 2% SDS, 5% β-mercaptoethanol with Bromophenol Blue). Samples were boiled before being subjected to SDS-PAGE on Mini-PROTEAN TGX Precast Gel 4-15% (Bio-Rad, 4561086). Western blotting using anti-GFP (Roche, 11814460001) and anti-PCNA (Santa Cruz Biotechnology, sc-56) antibodies was performed. For the analysis of cellular patterns of global SUMOylation, whole-protein extraction was performed as follows: aliquots of 2×10^8^ cells were collected and resuspended in 400 µl of water, then the cell suspensions were mixed with 350 µl of freshly prepared lysis buffer (2 M NaOH, 7% β-mercaptoethanol) and 350 µl of 50% TCA (Sigma-Aldrich, T9159). After spin, pellets were further washed with 1 M Tris pH 8 and resuspended in 2× Laemmli buffer (62.5 mM Tris pH 6.8, 20% glycerol, 2% SDS, 5% β-mercaptoethanol with Bromophenol Blue). Samples were boiled before being subjected to SDS-PAGE on Mini-PROTEAN TGX Precast Gel 4-15% (Bio-Rad, 4561086). Western blotting using anti-SUMO antibody (non-commercial, produced in rabbit by Agro-Bio) was performed (dilution of 1:1000).

### RT-qPCR

Total RNA was extracted from 1×10^7^ mid-log phase cells using the Masterpure Yeast RNA Purification Kit (Epicentre), according to the manufacturer's instructions. 1 µg of extracted RNA was treated with TURBO DNase (Ambion) for 1 h at 37°C, and reverse transcription was performed using random hexamers (Roche) and Superscript III reverse transcriptase (Invitrogen). Lightcycler 480 SYBR Green (Roche) and primers (_q_cen[dg]_F: 5′-AATTGTGGTGGTGTGGTAATAC-3′ and _q_cen[dg]_R: 5′-GGGTTCATCGTTTCCATTCAG-3′; _q_mat[D]_F: 5′-GTCCGAGGCAATACAACTTTGG-3′ and _q_mat[D]_R: 5′-GGTTGACAGTAGGAGATATTTACAG-3′; _q_act1_F: 5′-GTTTCGCTGGAGATGATG-3′ and _q_act1_R: 5′-ATACCACGCTTGCTTTGAG-3′) were used for qPCR quantification of pericentromere (*dg*) and mating-type locus (*mat*) transcript levels, relative to *act1^+^*.

### Statistical analysis

Quantitative analysis of western blots was carried out using Fiji software. The ratio from the Raw Integrated Density value of the protein of interest to housekeeping control was calculated for estimating the amount of protein.

Cell imaging was performed using METAMORPH software and processed and analyzed using ImageJ software (https://imagej.net/ij/). The explanation and definitions of values and error bars are mentioned within the figure legends. In most experiments, the number of samples is >2 and obtained from independent experiments to ensure biological reproducibility. For all experiments based on the analysis of cell imaging, the number of nuclei analyzed is mentioned in the figure legends. Statistical analysis was carried out using Mann–Whitney *U*-tests, Brown-Forsythe and Welch ANOVA test, chi-squared test and Student's *t*-test. Non-significant (ns), *P*≥0.05; *P≤0.05, **P≤0.01, ***P≤0.001, ****P≤0.0001.

## Supplementary Material

10.1242/biolopen.061746_sup1 Supplementary information
