# Supplementary material for: The fission yeast SUMO-targeted ubiquitin ligase Slx8 functionally associates with clustered centromeres and the silent mating-type region at the nuclear periphery
Source: Biol Open. 2024 Dec 30;13(12):bio061746. doi: 10.1242/bio.061746 (PMC11708773; doi:10.1242/bio.061746)
Supplement: Supplementary information [file biolopen-13-061746-s1.pdf]

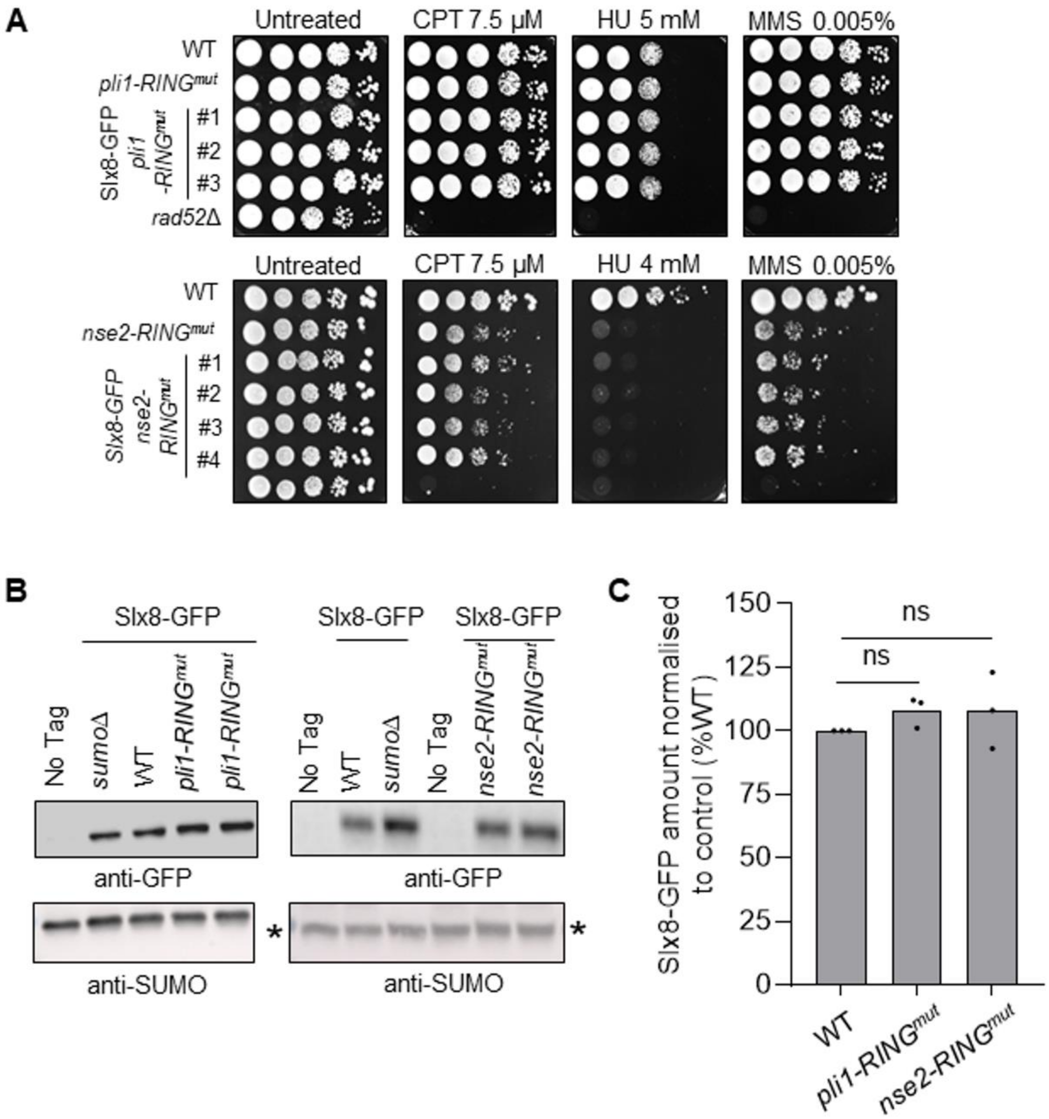

Fig. S1. Expression of Slx8-GFP is not affected by the absence of the E3 SUMO ligase activity of either Nse2 or Pli1.

A. Sensitivity of indicated strains to genotoxic drugs. Ten-fold serial dilution of exponential cultures were dropped onto indicated plates. HU: hydroxyurea; CPT: camptothecin and MMS: methyl methane sulfonate.

B. Expression of Slx8-GFP in indicated strains. An untagged WT strain (No Tag) was included as control for antibody specificity. An unspecific band (*) from SUMO-blots was used as a loading control.

Biology Open • Supplementary information

C. Quantification of Slx8-GFP expression in indicated strains. Dots represent values obtained from independent biological experiments. The normalized amount of Slx8 was calculated by dividing the GFP signal by unspecific SUMO signal. The normalized amount of Slx8-GFP in mutants was indicated as a percentage of WT. *p* value was calculated by two-sided Fisher's exact test (ns: non-significant).

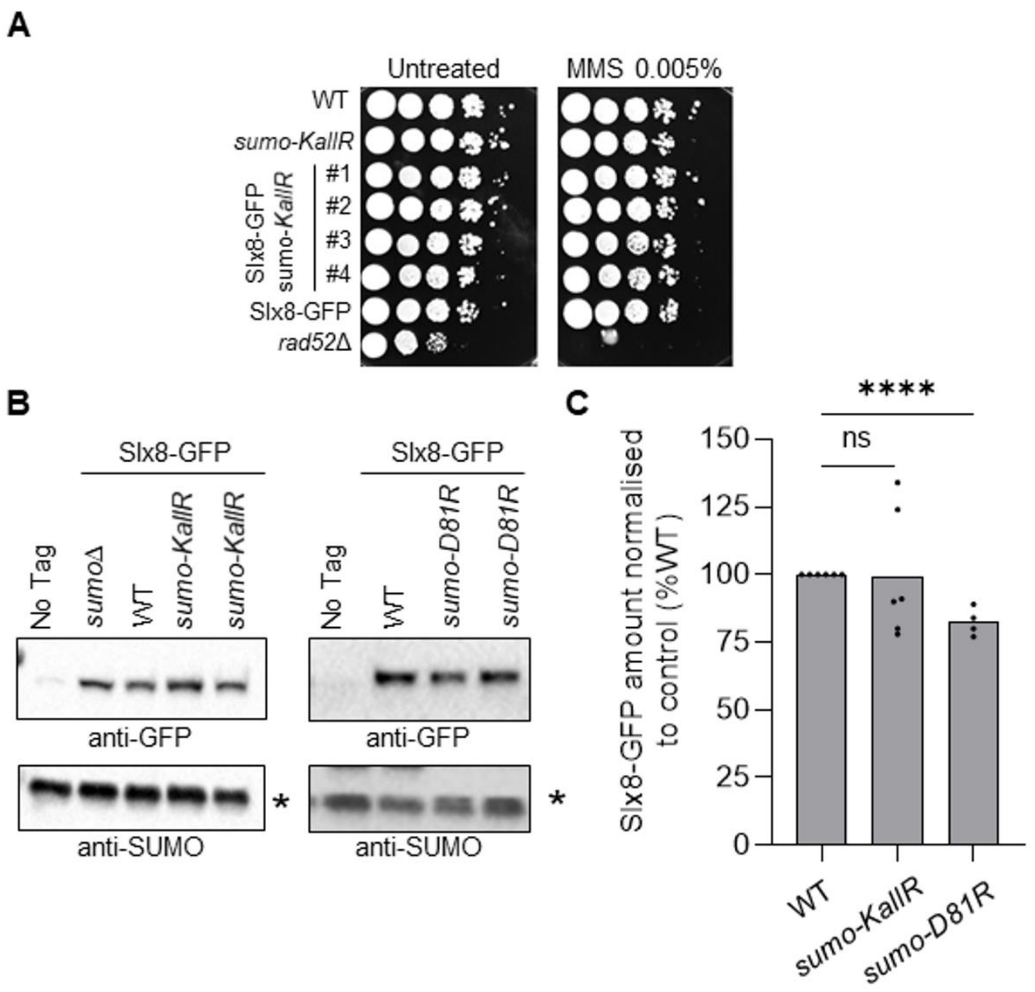

Fig. S2. Expression of Slx8-GFP is not affected in strains expressing SUMO-D81R or SUMO-KallR.

A. Sensitivity of indicated strains to genotoxic drugs. Ten-fold serial dilution of exponential cultures were dropped onto indicated plates. MMS: methyl methane sulfonate.

B. Expression of Slx8-GFP in indicated strains. An untagged WT strain (No Tag) was included as control for antibody specificity. An unspecific band (*) from SUMO-blots was used as a loading control.

C. Quantification of Slx8-GFP expression in indicated strains. Dots represent values obtained from independent biological experiments. The normalized amount of Slx8 was calculated by dividing the GFP signal by unspecific SUMO signal. The normalized amount of Slx8-GFP in mutants was indicated as a percentage of WT. *p* value was calculated by two-sided Fisher's exact test (**** p<0.0001; ns: non-significant).

Biology Open • Supplementary information

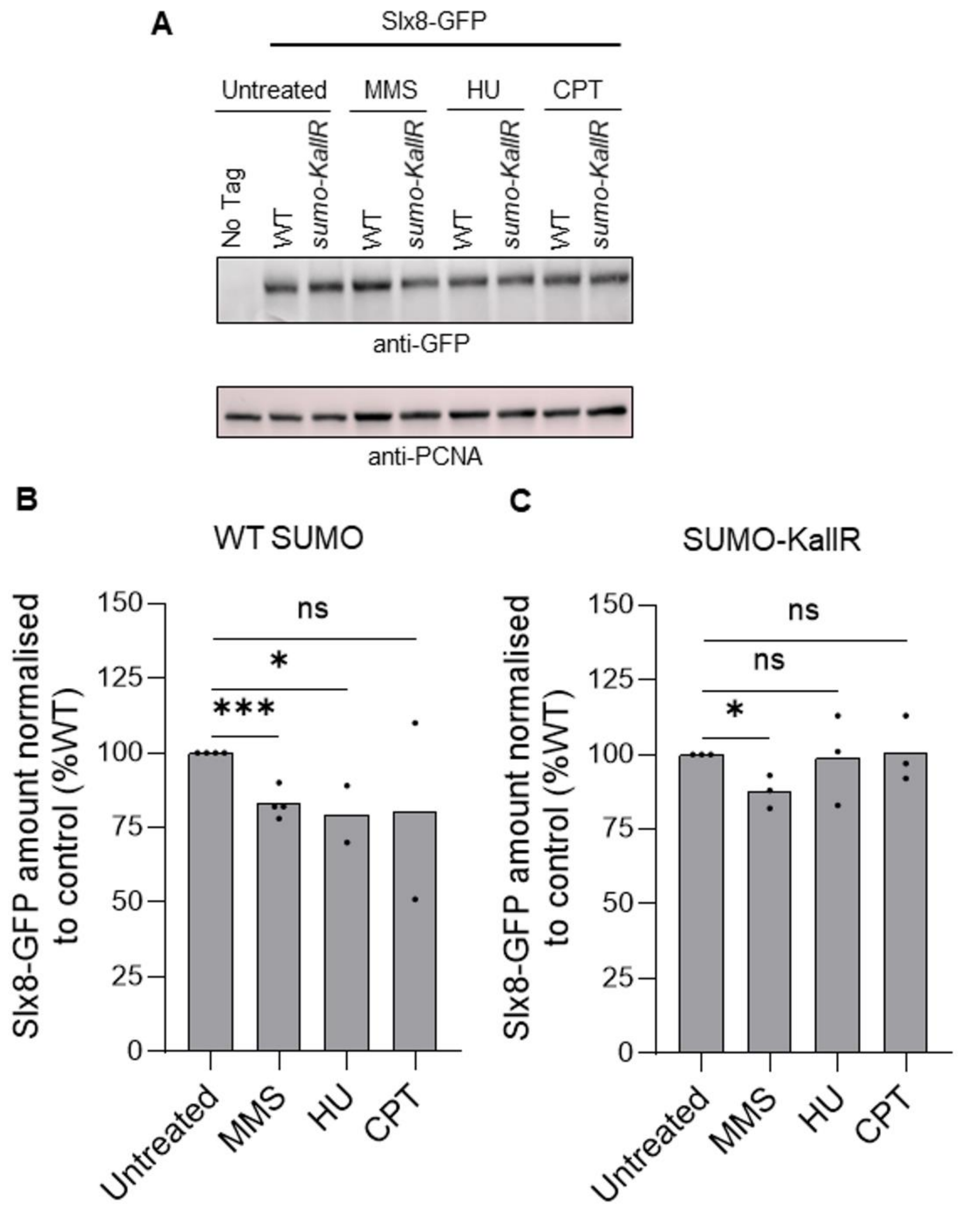

Fig. S3. Genotoxic treatments have a variable effect upon Slx8-GFP protein expression profile.

A. Expression of Slx8-GFP in indicated strains and conditions. An untagged WT strain (No Tag) was included as control for antibody specificity. PCNA was used as a loading control. HU: hydroxyurea; CPT: camptothecin and MMS: methyl methane sulfonate.

Biology Open • Supplementary information

B & C. Quantification of Slx8-GFP expression in indicated strains (WT SUMO: left panel, SUMO-KallR: right panel) and conditions. Dots represent values obtained from independent biological experiments. The normalized amount of Slx8 was calculated by dividing the GFP signal by PCNA signal. The normalized amount of Slx8-GFP in treated conditions was indicated as a percentage of the untreated conditions. *p* value was calculated by two-sided Fisher's exact test (*** $p \leq 0.001$; * $p \leq 0.05$; ns: non-significant).

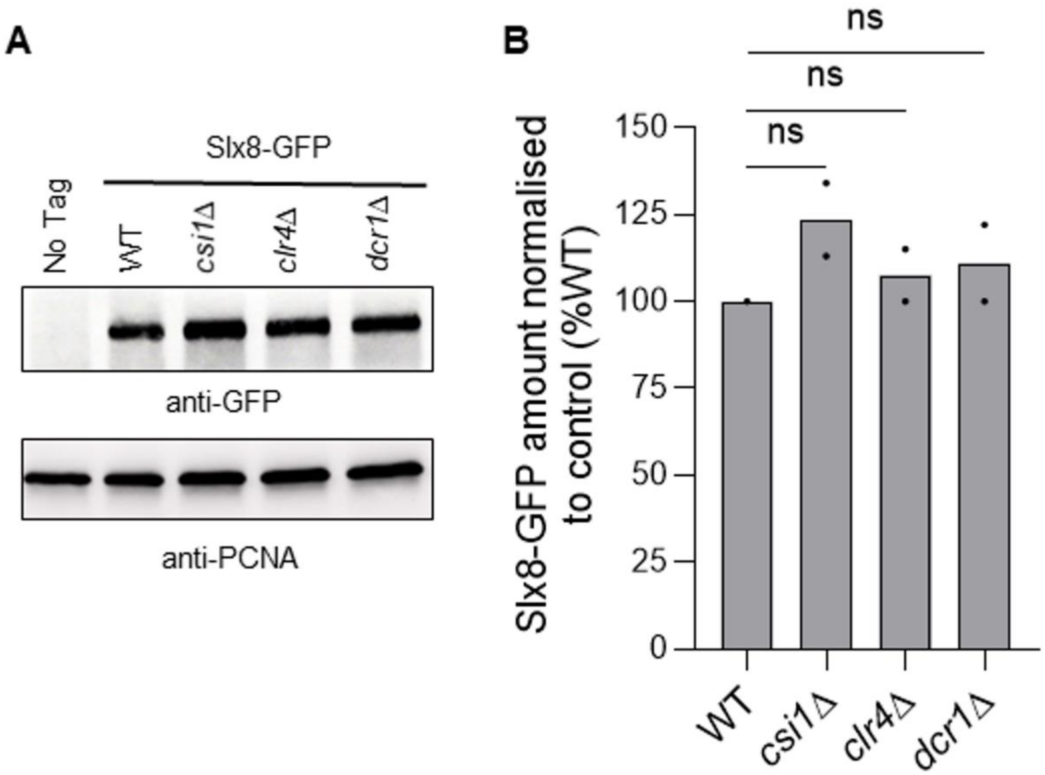

Fig. S4. Expression of Slx8-GFP is not affected by the absence of Clr4, Dcr1 or Csi1.

A. Expression of Slx8-GFP in indicated strains. An untagged WT strain (No Tag) was included as control for antibody specificity. PCNA was used as a loading control.

B. Quantification of Slx8-GFP expression. Dots represent values obtained from independent biological experiments. The normalized amount of Slx8 was calculated by dividing the GFP signal by PCNA signal. The normalized amount of Slx8-GFP in mutants was indicated as a percentage of the WT. *p* value was calculated by two-sided Fisher's exact test (ns: non-significant).

Table S1. Strains used in this study

Strain number	Mating type	Genotype	Reference
KK1492	*h-*	*slx8-GFP:natMX6 nmt41:rtf1:sup35 ade6-704 leu1-32 t-ura4-SD20<ori (uraR)*	this study
KK2021	*h+*	*slx8-GFP:natMX6 ade6-704 leu1-32 ura4-D18*	this study
KK1377	*h+*	*ade6-704 leu1-32 ura4-D18*	this study
KK772	*h+*	*rad52::kanMX6 nmt41:rtf1:sup35 ade6-704 t-*$ura4^{+}$*<ori (uraR) leu1-32*	this study
KK1562	*h-*	*pmt3::kanMX6-*$ura4^{+}$ *nmt41:rtf1:sup35 ade6-704 leu1-32 t-ura4-SD20<ori (uraR)*	this study
KK1025	*h-*	*slx8-29:hphMX6 nmt41:rtf1:sup35 ade6-704 leu1-32 t-ura4-SD20<ori (uraR)*	this study
KK2096	*h-*	*pli1-C321S-H323A-C326S (pli-*$RING^{mut}$*) slx8-GFP:natMX6 ade6-704 leu1-32 ura4-D18*	this study
KK2112	*h+*	*nse2-C195S-H197A (nse2-*$RING^{mut}$*) slx8-GFP:natMX6 ade6-704 leu1-32 ura4-D18*	this study
KK2023	*h+*	*pmt3-KallR (sumo-KallR) slx8-GFP:natMX6 ade6-704 leu1-32 ura4-D18*	this study
KK2074	*h-*	*pmt3-D81R (sumo-D81R) slx8-GFP:natMX6 ade6-704 leu1-32 ura4-D18*	this study
KK2176	*h+*	*slx8-GFP:natMX6 cut11-mCherry:hphMX6 ade6-704 leu1-32 ura4-D18*	this study
KK2173	*h+*	*pmt3-KallR (sumo-KallR) slx8-GFP:natMX6 cut11-mCherry:hphMX6 ade6-704 leu1-32 ura4-D18*	this study
KK2294	*h-*	*slx8-GFP:natMX6 sid4-mRFP:kanMX6 ade6-704 leu1-32 ura4-D18*	this study
KK2201	*h+*	*slx8-GFP:natMX6 mis6-mRFP:hphMX6 ade6-704 leu1-32 ura4-D18*	this study
KK2217	*h+*	*slx8-GFP:natMX6 taz1-mRFP:hphMX6 ade6-704 leu1-32 ura4-D18*	this study
KK2602	*h90*	*slx8-GFP:natMX6 arg3::mCherry-LacI his2::kanR-*$ura4^{+}$*-lacOp ade6-704 leu1-32 ura4-D18*	this study
KK2471	*h-*	*csi1::hphMX6 slx8-GFP:natMX6 ade6-704 leu1-32 ura4-D18*	this study
KK2432	*h+*	*clr4::natMX6 slx8-GFP:natMX6 ade6-704 leu1-32 ura4-D18*	this study

KK2436	*h-*	*dcr1::hphMX6 slx8-GFP:natMX6 ade6-704 leu1-32 ura4-D18*	this study
673	*h90*	*mat3-M:ade6^{+} ade6-DN/N leu1-32 ura4-D18*	this study
674	*h90*	*clr4Δ::leu2 mat3-M:ade6^{+} ade6-DN/N leu1-32 ura4-D18*	this study
6711	*h90*	*slx8Δ::ura4^{+} mat3-M:ade6^{+} ade6-DN/N leu1-32 ura4-D18*	this study
5513	*h90*	*Sid4^{+}-mRFP:Kan^{R} GFP-Cnp1^{+}:Nat^{R} leu1-32 ura4-D18*	this study
6363	*h+*	*csi1Δ::Hyg^{R} Sid4^{+}-mRFP:Kan^{R} GFP-Cnp1^{+}:Nat^{R} leu1-32 ura4-D18*	this study
7681		*slx8Δ::Kan^{R} Sid4^{+}-mRFP:Kan^{R} GFP-Cnp1^{+}:Nat^{R} leu1-32 ura4-D18*	this study
8000		*csi1Δ::Hyg^{R} slx8Δ::Kan^{R} Sid4^{+}-mRFP:Kan^{R} GFP-Cnp1^{+}:Nat^{R} leu1-32 ura4-D18*	this study
8028		*slx8Δ::Kan^{R} pli1Δ::ura4^{+} Sid4^{+}-mRFP:Kan^{R} GFP-Cnp1^{+}:Nat^{R} leu1-32 ura4-D18*	this study
7036		*pli1Δ::ura4^{+} Sid4^{+}-mRFP:Kan^{R} GFP-Cnp1^{+}:Nat^{R} leu1-32 ura4-D18*	this study

Biology Open • Supplementary information
